# Transarterial Embolization of Ruptured Pancreaticoduodenal Artery Pseudoaneurysm Related to Chronic Pancreatitis

**DOI:** 10.3390/diagnostics13061090

**Published:** 2023-03-14

**Authors:** Lucian Mărginean, Adrian Vasile Mureșan, Emil Marian Arbănași, Cătălin Mircea Coșarcă, Eliza Mihaela Arbănași, Eliza Russu, Rares Cristian Filep, Réka Kaller

**Affiliations:** 1 Department of Radiology, Mureș County Emergency Hospital, 540136 Targu Mures, Romania; 2 Doctoral School of Medicine and Pharmacy, George Emil Palade University of Medicine, Pharmacy, Sciences and Technology of Targu Mures, 540142 Targu Mures, Romania; emilarbanasi1@gmail.com (E.M.A.);; 3 Clinic of Vascular Surgery, Mures County Emergency Hospital, 540136 Targu Mures, Romaniaeliza.russu@umfst.ro (E.R.); 4 Department of Vascular Surgery, George Emil Palade University of Medicine, Pharmacy, Science, and Technology of Targu Mures, 540139 Targu Mures, Romania; 5 Department of Anatomy, George Emil Palade University of Medicine, Pharmacy, Science, and Technology of Targu Mures, 540139 Targu Mures, Romania; 6 Faculty of Pharmacy, George Emil Palade University of Medicine, Pharmacy, Science, and Technology of Targu Mures, 540139 Targu Mures, Romania

**Keywords:** pseudoaneurysms, embolization, pancreaticoduodenal artery, choils, CTA, PDA

## Abstract

We presented a 67-year-old woman with lightheadedness, diaphoresis, and acute epigastric and right hypochondrium pain, with a past medical history including stage 2 essential hypertension, chronic ischemic cardiomyopathy, and class 1 obesity. An abdominal contrast-enhanced CT scan showed an extensive hematoma (3 × 4 cm^2^ in size) located intra-abdominally, adjacent to the duodenojejunal area, with hyperdensity around the duodenum, positioned inferior to the pancreas (30–59 HU). Moreover, the CT scan also revealed an enhancing lesion as a pseudoaneurysm of the inferior pancreaticoduodenal artery, measuring 5 × 8 × 8 mm^3^ with active bleeding and associated hematoma. Following these investigations of the abdominal area, a decision was made to proceed with an endovascular intervention within the interventional radiology department. With the patient under conscious sedation, via a right common femoral artery approach, the superior mesenteric artery was catheterized. While injecting the contrast agent to obtain a better working projection, the pseudoaneurysm ruptured, and acute extravasation of the contrast agent was noted, followed by injection of a mixture of 1 mL Glubran 2 with 2 mL Lipiodol until complete obliteration of the pseudoaneurysm was obtained. The patient was hemodynamically stable at the end of the procedure and was discharged 6 days later in a good condition without active bleeding signs.

**Figure 1:**
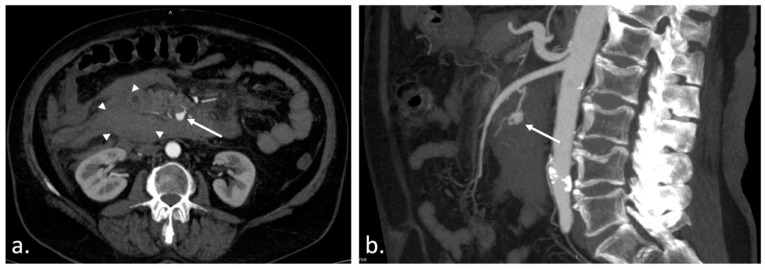
Aneurysmal dilatation of visceral arteries occurs in less than 1% of arterial aneurysm cases [1,2], primarily affecting the splenic artery, hepatic artery, and superior mesenteric artery; in only 1–2% of visceral aneurysms, it is found in the pancreaticoduodenal or gastroduodenal artery [1], with a high mortality rate in untreated cases [2,3,4]. Arterial aneurysmal dilatation develops as a result of damage to the collagen structure at the arterial wall caused by atherosclerosis or hypertension, and is found with the highest incidence at the level of the abdominal aorta [5,6,7,8,9]. In contrast, arterial pseudoaneurysms are connected with trauma, surgical treatments, or the presence of malignancies, but in the case of the pancreaticoduodenal artery, the main reason is the association with chronic pancreatitis [9,10,11,12,13,14,15,16]. Although it is a rare pathology, it is lethal in the absence of intervention. Endovascular treatment is currently the primary approach in the event of these pathologies, with a high success rate and excellent patient progression [4,9,13,14,15,16,17,18,19,20,21,22,23]. A 67-year-old woman presented with lightheadedness, diaphoresis, and acute epigastric and right hypochondrium pain. Along with other antecedents, her past medical history includes stage 2 essential hypertension, chronic ischemic cardiomyopathy, and class 1 obesity. Furthermore, three weeks before her current admission, she experienced a SARS-CoV-2 infection. She has no history of alcohol abuse, smoking, or abdominal trauma. An abdominal contrast-enhanced CT scan showed an extensive hematoma (3 × 4 cm^2^ in size) located intra-abdominally, adjacent to the duodenojejunal area, with hyperdensity around the duodenum and inferior to the pancreas (30–59 HU). Moreover, the CT scan also revealed an enhancing lesion as a pseudoaneurysm of the inferior pancreaticoduodenal artery measuring 5 × 8 × 8 mm^3^ with active bleeding and an associated hematoma. Additionally, the pancreas showed multiple hyperdense hemorrhagic structures anteriorly and superiorly under the aspect of possible acute hemorrhagic-ulcerative pancreatitis. Following these investigations of the abdominal area, a decision was made to proceed with an endovascular intervention within the interventional radiology department. (**a**) Transverse CT angiogram image shows a 12 mm enhancing lesion (white arrow) and a large intraperitoneal hematoma (arrowheads); (**b**) Sagittal MIP image depicts the enhancing lesion as a pseudoaneurysm (white arrow) of the inferior pancreaticoduodenal artery, responsible for the bleeding.

**Figure 2:**
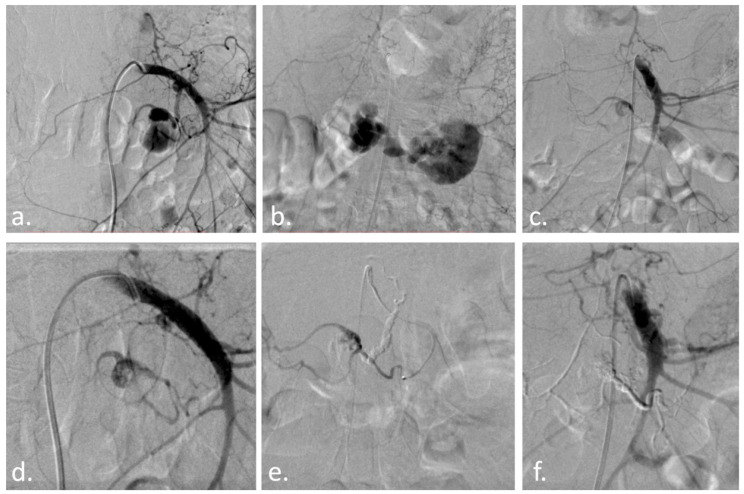
With the patient under conscious sedation, via a right common femoral artery approach, the superior mesenteric artery was catheterized with a 5F Cobra catheter and a Terumo 35 guidewire (Figure 2a). While injecting the contrast agent to obtain a better working projection, the pseudoaneurysm ruptured, and acute extravasation of the contrast agent was noted (Figure 2b). A Direxion microcatheter (Boston Scientific, Marlborough, MA, USA) with a 14 guidewire was selectively advanced in the inferior pancreaticoduodenal artery and another small pancreatic branch (Figure 2c–e), followed by injection of a mixture of 1 mL Glubran 2 (GEM, Viareggio, Italy) with 2 mL Lipiodol (Guerbet, Villepinte, France) until complete obliteration of the pseudoaneurysm was obtained (Figure 2f). The patient was hemodynamically stable at the end of the procedure and was discharged 6 days later in a good condition without any active bleeding signs. (**a**) Superior mesenteric artery contrast injection reveals an oval-shaped pseudoaneurysm; (**b**) Acute contrast extravasation during contrast injection; (**c**) Residual filling of the pseudoaneurysm through collaterals, despite IPD artery occlusion with glue; (**d**) Collateral branch responsible for residual filling; (**e**) Microcatheter in the inferior pancreaticoduodenal artery; (**f**) Complete obliteration of the pseudoaneurysm with the glue cast visible. Given the high morbidity and fatality rates associated with pancreaticoduodenal pseudoaneurysm rupture [23], immediate treatment is vital. Several recent studies in the literature demonstrate promising outcomes for endovascular embolization. Suzuki et al. [24] showed a series of seven patients embolized using micro-coils, with a 100% success rate and no long-term (28 months average, range 5–65 months) recurrence of symptoms or bleeding. Waguri et al. [23] also reported effective embolization of an anterior inferior pancreaticoduodenal artery pseudoaneurysm that perforated in the portal system. Furthermore, Krishna et al. [20] described a 60-year-old female patient who presented with melena and hematemesis caused by a rupture of the lower pancreaticoduodenal artery pseudoaneurysm at the level of the intestinal wall. Ren et al. [21] reported on their endovascular treatment of 159 patients with visceral artery aneurysms and pseudoaneurysms, with 96.9% of patients successfully treated and a 1.9% mortality rate at 30 days. Similar to our case, Mitrovic et al. [13,14,15] reported successful endovascular resolution of a posterior inferior pancreaticoduodenal artery pseudoaneurysm using the sandwich technique, in which the pseudoaneurysm inflow and outflow were embolized with coils. Jang et al. [16], on the other hand, embolized the pseudoaneurysm with an N-Butyl-Cyanoacrylate-lipiodol composition. Gurala et al. [17] also reported an effective embolization of a pseudoaneurysm from the gastro-duodenal artery, using coils. Inferior pancreaticoduodenal artery pseudoaneurysms may be diagnosed using ultrasound, CT, or visceral angiography with sensitivities of 50%, 67%, and 100%, respectively. An abdominal contrast-enhanced CT scan is usually sufficient for the appropriate identification of visceral pseudoaneurysms, but it should be confirmed through angiography and treated if necessary [13,14]. Treatments may involve direct thrombin injections, occlusive balloon catheters, surgical ligation, or percutaneous transcatheter embolization with coils or synthetic particles [9]. Vascular embolization with NBCA should proceed with extensive and careful evaluation of the vascular anatomy and close attention to technical details. NBCA is typically mixed with iodized oil (Lipiodol) for visualization under X-rays and to adjust the polymerization rate. Interventional radiologists should become more familiarized with these types of embolic agents, as they can be used as first-line treatment for various peripheral abnormalities [25]. Pseudoaneurysms of the visceral arteries are identified in up to 60% of cases at the level of the splenic artery, 25% of cases at the level of the renal arteries, and up to 20% of cases at the level of the hepatic artery. Additionally, with a considerably lower frequency of just under 5%, they are seen at the level of the celiac trunk, the mesenteric arteries, and in the smallest proportion at the level of the gastroduodenal and pancreaticoduodenal arteries [26,27,28,29,30,31,32,33,34,35,36,37,38,39,40,41,42]. Each patient with a visceral artery aneurysm should be treated individually and followed by a multidisciplinary team involving vascular and general surgeons, gastroenterologists, and radiologists for the best management choice. Endovascular therapy is the first option for these patients, based on our expertise and research published in the literature.
